# Potential therapeutic antibodies targeting specific adiponectin isoforms in rheumatoid arthritis

**DOI:** 10.1186/s13075-018-1736-3

**Published:** 2018-10-30

**Authors:** Yeon-Ah Lee, Dae-Hyun Hahm, Jung Yeon Kim, Bonjun Sur, Hyun Min Lee, Chun Jeih Ryu, Hyung-In Yang, Kyoung Soo Kim

**Affiliations:** 1 East-West Bone & Joint Disease Research Institute, Kyung Hee University Hospital at Gangdong, 02447 Seoul, Korea; 2 Division of Rheumatology, Department of Internal Medicine, College of Medicine, Kyung Hee University, 23 Kyung Hee Dae-ro, Dongdaemun-gu, 02447 Seoul, Korea; 3 Department of Physiology, College of Medicine, Kyung Hee University, 23 Kyung Hee Dae-ro, Dongdaemun-gu, 02447 Seoul, Korea; 4 Department of Pathology, Inje University Sanggye Paik Hospital, 1342 Dongil-ro, Nowon-gu, 01757 Seoul, Korea; 5 Acupuncture and Meridian Science Research Center, College of Korean Medicine, Kyung Hee University, 23 Kyung Hee Dae-ro, Dongdaemun-gu, 02447 Seoul, Korea; 6 Department of Integrative Bioscience and Biotechnology, Sejong University, 209 Neungdong-ro, Gwangjin-gu, 05006 Seoul, Korea; 7 Department of Clinical Pharmacology and Therapeutics, College of Medicine, Kyung Hee University, 23 Kyung Hee Dae-ro, Dongdaemun-gu, 02447 Seoul, Korea

**Keywords:** Adiponectin isomer, Monoclonal antibody, Hybridoma, Rheumatoid arthritis, CIA (collagen-induced arthritis) mouse model, Therapeutic antibody

## Abstract

**Background:**

Different adiponectin isoforms appear to be differentially involved in the pathogenesis of various diseases. The purpose of this study was to generate monoclonal antibodies (mAbs) specific to different adiponectin isoforms and investigate whether these mAbs have potential as therapeutic agents for such diseases.

**Methods:**

Hybridoma cells producing monoclonal antibodies were generated and screened using enzyme-linked immunosorbent assay and Western blotting for the production of mAbs recognizing human adiponectin isoforms.

**Results:**

The mAb from hybridoma clone KH7–41 recognized both the middle molecular weight (MMW) (hexamer) and low molecular weight (LMW) (trimer) isoforms of adiponectin in human serum, whereas the KH7–33 mAb detected only MMW (hexamer) adiponectin. The KH4–8 clone recognized both the high molecular weight (HMW) (multimer) and MMW adiponectin isoforms. However, in mouse and rat sera, the abovementioned antibodies recognized only the MMW isomer. These mAbs also recognized adiponectin in various human tissues, such as lung, kidney, and adipose tissues, although the three mAbs had different staining intensities. The mAb from clone KH4–8 effectively inhibited increases in interleukin-6 (IL-6) and IL-8 expression in recombinant adiponectin-stimulated human osteoblasts and human umbilical vein endothelial cells. Also, the mAbs KH7–33 and KH4–8 significantly ameliorated rheumatic symptoms in a collagen-induced arthritis mouse model. This result suggests that these mAb treatments may ameliorate adiponectin-mediated inflammatory response.

**Conclusions:**

mAbs against human adiponectin isomers can potentially be developed as therapeutic antibodies to target specific detrimental isoforms of adiponectin while maintaining the functions of beneficial isoforms.

**Electronic supplementary material:**

The online version of this article (10.1186/s13075-018-1736-3) contains supplementary material, which is available to authorized users.

## Background

Adipose tissue produces a variety of adipokines (leptin, adiponectin, resistin, and visfatin) as well as pro- and anti-inflammatory cytokines (tumor necrosis factor-alpha (TNF-α), interleukin-4 [IL-4] and IL-6, and others) [1]. Thus, adipose tissue, though once viewed as simply a lipid storage and release depot, is now considered an endocrine tissue [2]. Among adipokines, adiponectin seems to be involved in the pathogenesis of various diseases [3, 4]. In particular, adiponectin levels in synovial fluid and serum are elevated in patients with rheumatoid arthritis (RA) [5, 6]. Adiponectin also induces the production of pro-inflammatory cytokines IL-6, matrix metalloproteinase-1 (MMP-1), and IL-8/CXCL8 by RA synovial fibroblasts *in vitro* [7, 8]. Furthermore, adiponectin stimulates osteopontin production in RA synovial tissue, which is required for osteoclast recruitment and contributes to bone erosion [9]. Expression of a pro-inflammatory cytokine, oncostatin, was also induced by adiponectin in osteoblasts. In a collagen-induced arthritis (CIA) mouse model, adiponectin exacerbated arthritis progression through enhancement of the T helper 17 (Th17) response and receptor activator of nuclear factor-kappa Β ligand (RANKL) expression [10]. In contrast, adiponectin has been suggested to have anti-inflammatory effects in the context of arthritis [11–13]. Thus, its exact role remains controversial. We recently suggested that adiponectin may contribute to synovitis and joint destruction in RA by stimulating the expression of vascular endothelial growth factor (VEGF) and MMP-1 and MMP-13 in fibroblast-like synoviocytes (FLSs) to a greater extent than do pro-inflammatory mediators [14]. In addition, at physiological concentrations, adiponectin has been suggested to be more important than IL-1β in stimulating the production of mediators that drive synovitis and joint destruction in endothelial cells and osteoblasts [15]. More importantly, we demonstrated that adiponectin in combination with IL-1β may have synergistic effects on the production of pro-inflammatory mediators during arthritic joint inflammation [16]. A recombinant adiponectin monomer produced in *Escherichia coli* was used in most of the above studies.

Adiponectin comprises a carboxyl-terminal globular domain and an amino-terminal collagenous domain [17]. It belongs to the soluble collagen superfamily and is structurally homologous to collagen VIII and X, complement factor C1q [18], and the TNF family [19]. Adiponectin belongs to a family of proteins that form characteristic multimers [20]. Using SDS-PAGE (sodium dodecyl sulfate-polyacrylamide gel electrophoresis) under non-reducing and non-heat-denaturing conditions, Waki et al. showed that adiponectin exists in a wide range of multimeric complexes in plasma and combines via its collagen domain to create three main oligomeric forms: a low-molecular-weight (LMW) trimer, a middle-molecular-weight (MMW) hexamer, and a high-molecular-weight (HMW) 12- to 18-mer [21]. These adiponectin isoforms seem to affect gene expression differently. Frommer et al. showed the differential effects of adiponectin isoforms on effector cells involved in RA pathophysiology: HMW/MMW-enriched and globular adiponectin strongly activated expression of chemokines and pro-inflammatory cytokines in RA synovial fibroblasts (RASFs), while the adiponectin trimer (LMW) led to minimal chemokine and cytokine expression [22]. In addition, adiponectin isoforms differentially affected lipid gene expression in primary human hepatocytes (PHHs) [23]. Population-based studies revealed that HMW adiponectin was negatively associated with low-density lipoprotein cholesterol, triglycerides, apolipoprotein B, and apolipoprotein E and was positively associated with high-density lipoprotein cholesterol [24–26]. Adiponectin isoforms also function as acute-phase reactants influencing inflammation in acute and chronic diseases. In obesity, adiponectin isoform formation is disrupted, leading to the development of pathologic conditions [27]. Given their pathophysiological effects, detrimental adiponectin isoforms could plausibly be targeted as a therapeutic strategy while maintaining the beneficial activities of other adiponectin isoforms. Here, we show that monoclonal antibodies (mAbs) against adiponectin isomers recognize adiponectin isoforms in sera and tissues and we demonstrate anti-arthritic effects in a CIA mouse model.

## Methods

### Hybridoma production and monoclonal antibody purification

Two BALB/c mice were immunized subcutaneously with 100 μL of complete Freund’s adjuvant (CFA) (Difco Laboratories, Detroit, MI, USA) containing 100 μg of recombinant human adiponectin expressed in *E. coli* (ProSpec, Rehovot, Israel). After 2 weeks, the mice were injected with incomplete Freund’s adjuvant. The mice were boosted with antigen only (that is, 50 μg of adiponectin intravenously) 2 weeks later. Two days after the last boost, sera were tested for reactivity to recombinant adiponectin using enzyme-linked immunosorbent assay (ELISA). Splenic lymphocytes were fused to FO myeloma cells (ATCC, Manassas, VA, USA) and plated on 96-well plates in Dulbecco’s modified Eagle’s medium (DMEM) supplemented with 20% fetal bovine serum (FBS) (Invitrogen, Waltham, MA, USA) and HAT component (Sigma-Aldrich Korea, Yongin, Korea) as described previously [28]. The culture supernatants were tested by Western blot and ELISA for reactivity to recombinant human adiponectin. mAbs were purified from culture supernatants of the screened clones by using Protein G-Sepharose column chromatography (GenScript, Piscataway, NJ, USA) in accordance with the protocol of the manufacturer. Studies were conducted in accordance with the National Institutes of Health guidelines and were approved by the Institutional Animal Care and Use Committee of Kyung Hee University.

### Collagen-induced arthritis mouse model

Male DBA/1 J mice (4 weeks old) were purchased from Central Lab. Animal Inc. (Seoul, Korea). The animals were kept in a rodent facility and adapted for at least 1 week before CIA induction as previously described [29]. Briefly, the mice (6 weeks old) were immunized at the base of the tail with a 100-μL mixture of chicken collagen type II (CII) 100 μg (Sigma-Aldrich Korea) and CFA. After 14 days, the mice were given a booster injection of 100 μg CII and incomplete Freund’s adjuvant. The mice were divided into five groups (n = 8) containing normal, CIA (saline, control), CIA + KH4–8 mAb (6 mg/kg, intraperitoneal injection, three times a week for 6 weeks), CIA + KH7–33 mAb (6 mg/kg, intraperitoneal injection, three times a week for 6 weeks), and CIA + prednisolone (10 mg/kg, intragastric administration, twice a week for 6 weeks). Normal mice and CIA control mice were given an equal volume of normal saline. The treatment antibody concentration was decided on the basis of the treatment dose for therapeutic antibodies in mice [30, 31]. To evaluate the therapeutic effect of mAbs KH4–8 and KH7–33 on the progression of arthritis in CIA mice, body weight, paw volume, squeaking score, and arthritic score were measured [29]. All methods were approved by the Animal Care and Use Committee of Kyung-Hee University (KHUASP[SE]-15–115). All procedures were executed in accordance with the guide for the Care and Use of Laboratory Animals by the Korea National Institute of Health.

### Cell culture for *in vitro* functional testing of monoclonal antibodies

Human umbilical vein endothelial cells (HUVECs) and human osteoblasts were obtained from the Korean Cell Line Bank (KCLB, Seoul, Korea) and Cell Applications, Inc. (San Diego, CA, USA), respectively. Endothelial cells and osteoblasts were cultured in T-75 flasks (Nunc, Thermo Fisher Scientific, Waltham, MA, USA) containing EGM-2 (Lonza, Alpharetta, GA, USA) and osteoblast growth medium (Cell Applications, Inc. San Diego, CA, USA), respectively. After the cells had grown to confluence, they were split at a 1:4 ratio. Cell passages 5–6 were used for all experiments. HUVECs (2 × 10^5^ cells per six-well plate in 2 mL of medium) and osteoblasts (1 × 10^5^ cells per six-well plate in 2 mL of medium) were cultured overnight and treated with human recombinant adiponectin, which was produced by using *E. coli* (ProSpec, Rehovot, Israel ). To test the ability of the mAb to block adiponectin function, mAb (*~*120 μg/mL) and adiponectin (2.5 or 5 μg/mL) were mixed and incubated for 1 h before being used to treat cells. After 24-h treatment, the culture supernatants were collected and frozen, and IL-6 and IL-8 were measured by ELISA.

### Epitope mapping of monoclonal antibody

Epitope mapping of the KH4–8 mAb was performed by using PEPperMAP^®^ technology (PEPperPRINT GmbH, Heidelberg, Germany) [32]. PEPperMAP^®^ Linear Epitope Mapping of mouse mAb KH4–8 was performed against human adiponectin translated into linear 15–amino acid peptides with a peptide-peptide overlap of 14 amino acids. Human adiponectin peptide microarrays were incubated with the mouse mAb at concentrations of 1 μg/mL, 10 μg/mL, and 100 μg/mL in incubation buffer followed by staining with secondary goat anti-mouse IgG (H + L) DyLight680 antibody. Samples were processed by using an Odyssey Imaging System (LI-COR, Lincoln, NE, USA). Quantification of spot intensities and peptide annotation were performed using a PepSlide^®^ Analyzer (PEPperPRINT GmbH).

### Western blotting

To screen hybridomas for production of mAbs against recombinant human adiponectin, adiponectin (100 ng/well) was resolved via 12% SDS-PAGE and transferred to Hybond-ECL membranes (Amersham, Arlington Heights, IL, USA). The membranes were blocked with 6% non-fat milk dissolved in TBST buffer (10 mM Tris-Cl [pH 8.0], 150 mM NaCl, 0.05% Tween 20). The blots were probed with hybridoma culture supernatants at 4 °C overnight and incubated with a 1:1,000 dilution of horseradish peroxidase–conjugated goat anti-mouse IgG secondary antibody (Sigma-Aldrich Korea). The blots were developed by using the ECL method (Amersham). To detect adiponectin in human, mouse, and rat serum samples, serum was subjected to gradient SDS-PAGE (4–12%) (NuPAGE^®^ Bis-Tris Mini Gels, Invitrogen), blotted, and probed with purified mAb (5 μg/mL) as the primary antibody, as described above. Mouse and rat serum were obtained via heart puncture of male BALB/c mice and Sprague Dawley (SD) rats (8 weeks old), respectively. Human serum was obtained from a male volunteer (55 years old).

### Enzyme-linked immunosorbent assay

Culture supernatants from cells treated with mAb plus adiponectin were analyzed with IL-6 and IL-8 ELISA kits (BD Bioscience Korea, Seoul, Korea) in accordance with the protocol of the manufacturer. To screen hybridoma clones for mAbs against adiponectin, 96-well plates were coated with adiponectin (200 ng/well), hybridoma culture supernatants were added after blocking, wells were incubated with a 1:1,000 dilution of horseradish peroxidase–conjugated goat anti-mouse IgG secondary antibody (Sigma-Aldrich Korea), and the ELISA procedure was completed. Sera from CIA were obtained from heart puncture and analyzed for adiponectin, IL-6, TNF-α, and RANKL by using the Luminex^®^ 200™ Total System (Luminex Corporation, Austin, TX, USA) as previously described [33].

### Histological assessment of inflammation

Mice were killed after 56 days of CII + CFA treatments. Histochemical staining was performed to determine the degree of immune cell infiltration into the joints. Mice knee joints were dissected, fixed for 3 days in 10% formalin, dehydrated through a graded ethanol series, cleared in xylene, and processed for embedding in paraffin wax with routine protocols. Coronal sections 8 mm thick were cut through the knee joint by using a manual rotary microtome (Finesse 325, Thermo Shandon Inc., Pittsburgh, PA, USA) and stained with hematoxylin and eosin for routine histological evaluation. Paraffin tissue sections obtained from rat knees were deparaffinized in xylene. The tissue samples were hydrated with ethanol and washed in distilled water, followed by antigen retrieval via heating with 100 mM citrate buffer (pH 6.0) at 65 °C for 1–2 h. The samples were examined with a confocal laser scanning microscope (Olympus BX53, Olympus Corporation, Tokyo, Japan). The degree of inflammation was evaluated on a scale from 0 to 4 by three different experts who were blinded to treatment information. The scale was defined as follows: 0 = no inflammation, 1 = minimal inflammation, 2 = mild inflammation, 3 = moderate inflammation, and 4 = severe inflammation.

### Immunohistochemistry

Normal human tissues were obtained from archived paraffin collections at the Department of Pathology, Inje University Sanggye Paik Hospital (Seoul, Korea). Adipose, lung, kidney, and pancreas tissues were obtained from different donors (a 59-year-old woman, a 72-year-old man, a 62-year-old woman, and a 67-year-old man, respectively). Sections (4 μm) of the paraffin blocks were cut and immunohistochemically stained with mAbs (50 μg/mL) by using an automated system (Vision BioSystems Ltd., Mount Waverley, Australia), as described below. Antigen was retrieved with epitope retrieval solution 1 (Leica Microsystems, Newcastle, UK). Slides were incubated with antibody at room temperature for 20 min and then with a biotinylated secondary antibody for 8 min. The resulting complexes were detected with avidin-peroxidase-conjugated polymer. Color was developed by using 3,3′-diaminobenzidine (DAB) (ScyTek, Logan, UT, USA). Mayer’s hematoxylin was used as a counterstain. Positive and negative control stains were used in each run. The study protocol was reviewed and approved by the institutional review board at Inje University Sanggye Paik Hospital. For immunohistochemical staining of adiponectin in the joints of mice using anti-adiponectin antibody (Abcam, Cambridge, MA, USA), moderate nuclear or cell membranous staining was determined as a percentage and scored as follows: 0 = staining in less than 10% of cells, 1 = staining in 10–50% of cells, and 2 = staining in more than 50% of cells. Cases with a score of 1 or 2 were classified as positive.

### Statistical analysis

*In vitro* data are expressed as the mean ± standard error of the mean of quadruplicate samples. The expression levels of the factors were compared between groups by using the Mann–Whitney test. Prism 5.02 software (GraphPad Software, San Diego, CA, USA) was used for statistical analysis and graphing. Statistical differences between CIA mouse groups were identified by using *t* tests, one-way analysis of variance (ANOVA) with Dunn’s Multiple Comparison Test, and two-way ANOVA followed by Bonferroni post-test correction (for multiple comparisons of body weight, squeaking score, paw volume, and arthritis index score). Differences were considered significant at a *P* value of less than 0.05.

## Results

### Screening hybridoma clones producing mAb against adiponectin isomers and mAb purification from culture supernatants

Each hybridoma clone generated against recombinant adiponectin was screened by using ELISA performed on its culture supernatant. The optical densities (ODs) of culture supernatants from positive hybridoma clones were 10 times higher than the ODs of negative clones (data not shown). The reactivity of mAb from each clone was confirmed by Western blotting. Hybridomas producing mAb against recombinant human adiponectin were subcloned and screened by using ELISA and Western blotting. To purify mAb from culture supernatants, supernatant (500 mL) from each hybridoma clone was passed through a Protein G-Sepharose column (2-mL bed volume), washed, and eluted with elution buffer in accordance with the protocol of the manufacturer. Approximately 15 mg of mAb was obtained from 500 mL of supernatant. mAb purity was determined by Coomassie staining after PAGE. Purified mAb was detected with anti-mouse IgG-HRP, which suggests that the protein purified from each supernatant was IgG (data not shown).

### Differences in patterns of recognition of adiponectin isoforms between the purified mAbs in serum

**Fig. 1:**
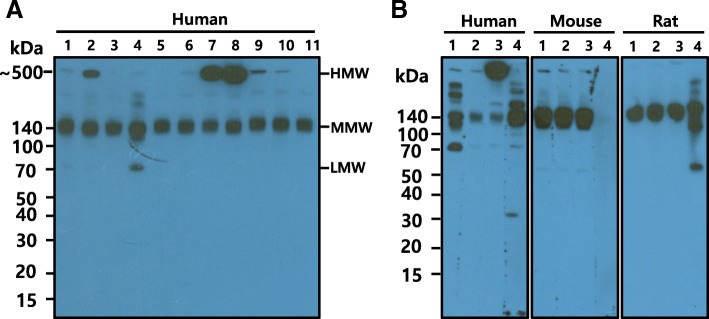
Detection of serum adiponectin isoforms with monoclonal antibodies (mAbs) against recombinant human adiponectin. **a** Western blot to screen mAbs detecting different adiponectin isoforms. Human serum was loaded into each lane of the gel and resolved by polyacrylamide gel electrophoresis (PAGE) and then transferred to a membrane. Each lane of the membrane was then separately cut and incubated with each mAb from hybridoma culture supernatant. After the secondary antibody was probed, the cut membrane was combined to form one sheet and developed with ECL solution. Eleven different mAbs from culture supernatant were tested to investigate their recognition patterns of adiponectin isoforms in serum. The mAb KH7–33, in lane 3, was selected as a representative recognizing only the middle molecular weight (MMW) isoform of adiponectin. The mAb KH7–41, in lane 4, was selected as a representative recognizing both the MMW and low molecular weight (LMW) isoforms of adiponectin. The mAb KH4–8, in lane 8, was selected as a representative mAb recognizing both the MMW and HMW isoforms of adiponectin. **b** Comparison of the mAb recognition patterns of adiponectin isoforms in human, mouse, and rat sera by Western blot. Human, mouse, and rat sera were separated by sodium dodecyl sulfate–PAGE. All three mAbs (KH7–33, KH7–41, and KH4–8) recognized the rat and mouse adiponectin MMW isoform. Lane 1, KH7–41; lane 2, KH7–33; lane 3, KH4–8; lane 4, commercial antibody against human adiponectin (Boster Immunoleader cat. no. PB9001) or mouse/rat adiponectin (Boster Immunoleader cat. no. PB9011). Mouse and rat serum were obtained via heart puncture of male BALB/c mice and Sprague Dawley rats (8 weeks old), respectively. Human serum was obtained from a male volunteer (55 years old)

To determine whether these purified mAbs recognized adiponectin isoforms in human serum, we analyzed the sera by Western blotting following gradient SDS-PAGE (4–12%). As shown in Fig. 1a, different mAbs recognized the various adiponectin isoforms in human serum to differing degrees. In particular, the mAb from KH7–41 (lane 4) recognized the trimeric (LMW) and hexameric (MMW) isoforms of human serum adiponectin. However, the mAb from KH7–33 (lane 3) detected only the hexameric (MMW) isoform whereas the KH4–8 mAb (lane 8) strongly recognized the HMW isoform of human adiponectin but weakly recognized the MMW isoform. The recognition patterns of adiponectin isoforms by these three mAbs in mouse and rat serum were compared with those in human serum (Fig. 1b). Unlike with human serum, the KH7–41 and KH4–8 mAbs did not recognize LMW or HMW adiponectin in mouse or rat serum. These mAbs recognized only MMW adiponectin in mouse and rat serum.

### Recognition of adiponectin isoforms in tissues using purified mAbs

**Fig. 2:**
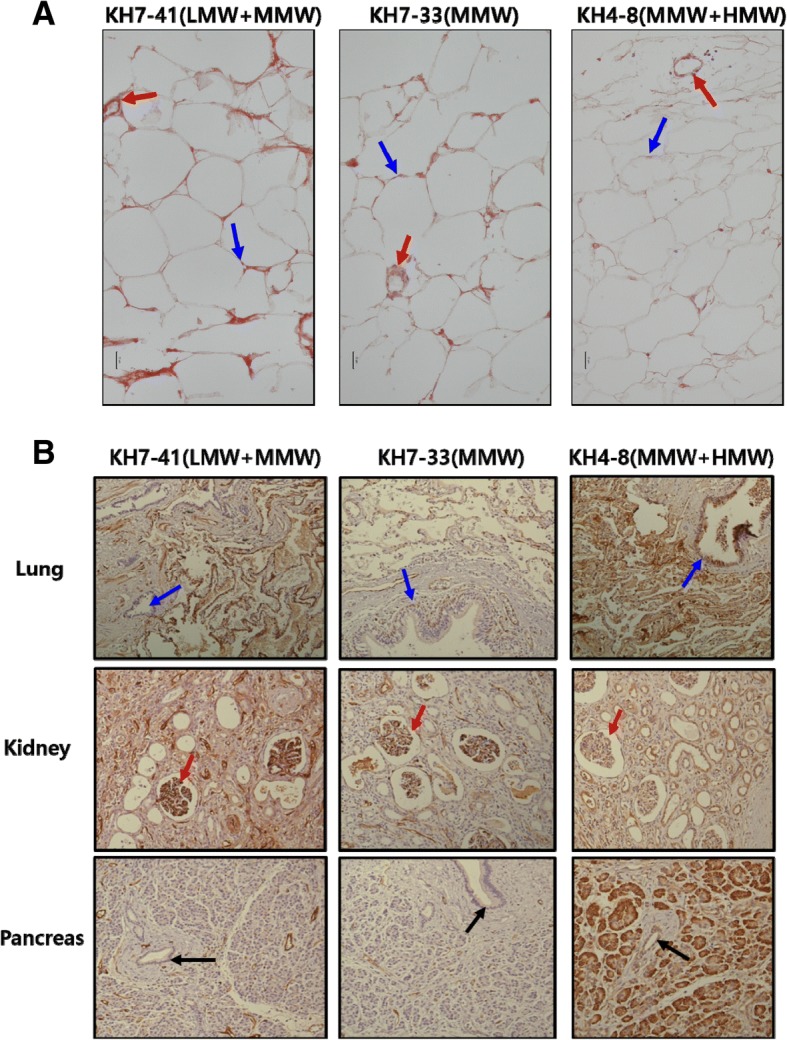
Immunohistological assays in human tissues with monoclonal antibodies (mAbs). To determine the pattern of recognition of adiponectin isoforms in human tissues by mAbs, **a** normal human adipose tissue was immunostained with mAbs (KH7–41, KH7–33, and KH4–8) (200×, scale bar = 25 μm). mAbs recognized adiponectin in the nucleus of adipocytes (blue arrow) and in vessels and endothelial cells (red arrow). **b** Human lung, kidney, and pancreas were stained with the KH7–41, KH7–33, and KH4–8 mAbs (100×). Abbreviations: *HMW* high molecular weight, *LMW* low molecular weight, *MMW* middle molecular weight

To test whether the three mAbs recognized adiponectin isoforms in tissues, human adipose tissue was incubated with each mAb (Fig. 2). All three mAbs stained adipose tissue. The staining patterns of the mAbs were not significantly different from one another. These three antibodies all recognized adiponectin both in the nuclei of adipocytes (blue arrow in Fig. 2a) and in blood vessels (red arrow in Fig. 2a). In contrast, other human tissues were differentially stained by different mAbs (Fig. 2b). In lung tissues, both KH7–41 and KH7–33 recognized adiponectin only in alveoli but not in bronchioles (blue arrow). KH4–8 recognized it in both alveoli and bronchioles, suggesting that the HMW isoform is predominant in bronchioles. In kidney tissue, KH7–33 recognized adiponectin only in glomeruli whereas both KH7–41 and KH4–8 recognized it in glomeruli and tubules. This suggests that the MMW isoform may be absent, or may be present only at a very low level, in tubules. In pancreatic tissue, both KH7–41 and KH7–33 recognized adiponectin only in vessels (yellow arrow) but not in acini or ducts (black arrow). However, KH4–8 recognized adiponectin in vessels, ducts, and acini, suggesting that the HMW isoform is present in all pancreatic tissue.

### Blocking of *in vitro* adiponectin activity by monoclonal antibodies

**Fig. 3:**
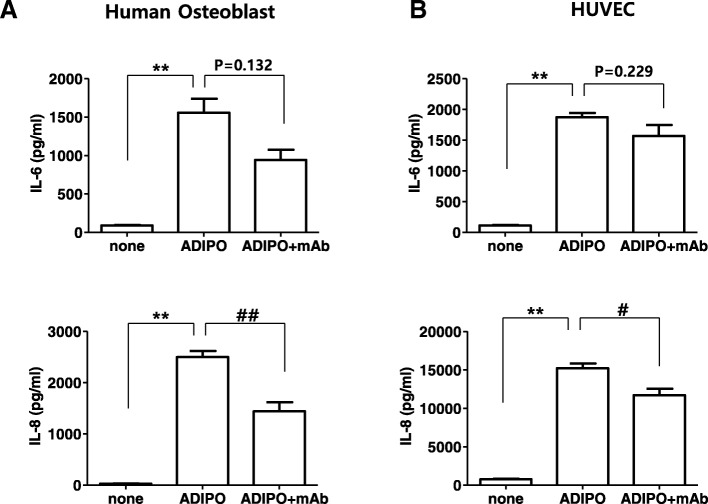
Inhibition of adiponectin-mediated gene expression *in vitro* by monoclonal antibodies (mAbs). To test the ability of the mAb KH4–8 to block adiponectin function, (**a**) human osteoblasts and (**b**) human umbilical vein endothelial cells (HUVECs) were treated with adiponectin (ADIPO) or KH4–8 mAb (mAb) or both. The mAb (~120 μg/mL) and recombinant adiponectin (2.5 μg/mL) were mixed and incubated for 1 h before being used to treat cells. After 24-h treatment, the culture supernatants were collected and frozen, and interleukin-6 (IL-6) and IL-8 were measured by using enzyme-linked immunosorbent assay (ELISA) (R&D Systems, Minneapolis, MN, USA). The experiments were performed in quadruplicate. The data shown are representative of three independent experiments, and similar results were obtained with all three mAbs. Values are expressed as mean ± standard error of the mean. The expression levels of the factors were compared between groups by using the Mann–Whitney test. *P <0.05, **P <0.01 versus the untreated group, ^#^*P* <0.05, ^##^*P* <0.01 versus the group treated with adiponectin and mAb

To test the biological activity of the purified mAbs, HUVECs and human osteoblasts were treated with adiponectin or mAbs or both. As shown in Fig. 3, treating human osteoblasts with adiponectin (2.5 μg/mL) significantly increased IL-6 and IL-8 production compared with levels in the untreated group. Combined administration of KH4–8 mAb (*~*120 μg/mL) and adiponectin (2.5 μg/mL) significantly inhibited this increase in IL-8 production but did not significantly inhibit the increase in IL-6 production. In addition, as was observed in osteoblasts, treating HUVECs with adiponectin (5 μg/mL) significantly increased IL-6 and IL-8 production compared with levels in the untreated group. Combined administration of KH4–8 mAb (*~*120 μg/mL) and adiponectin (5 μg/mL) significantly inhibited the increase in IL-8 production but did not significantly inhibit the increase in IL-6 production. Administration of other mAbs yielded inhibition similar to that seen with KH7–33 treatment (data not shown). These results suggest that these mAbs partially inhibit the activity of recombinant adiponectin *in vitro*.

### Epitope mapping of monoclonal antibody KH4–8

**Fig. 4:**
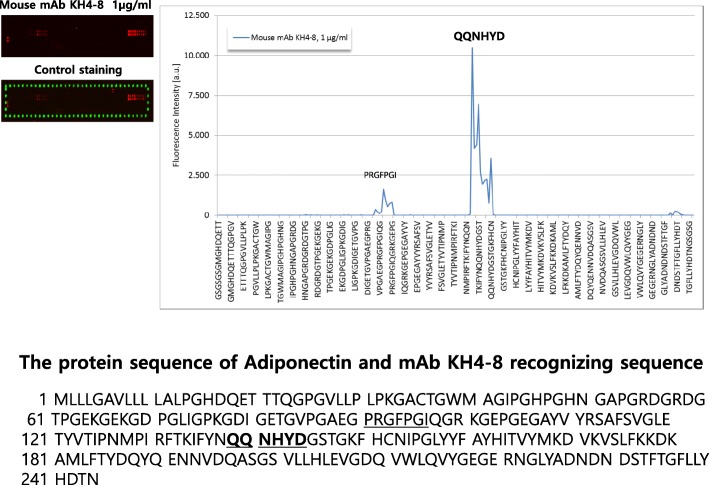
Epitope mapping of monoclonal antibody (mAb) KH4–8 against adiponectin. To identify the epitope-recognizing site of the KH4–8 mAb, PEPperMAP^®^ technology was performed as described in the Methods. Human adiponectin was translated into linear 15–amino acid peptides with a peptide-peptide overlap of 14 amino acids. Human adiponectin peptide microarrays were incubated with mouse mAb KH4–8 at different concentrations followed by staining with secondary goat anti-mouse IgG (H + L) DyLight680 antibody. The light intensity was read by a reader. The amino acid sequence QQNHYD (139–144) was confirmed from among the full 244–amino acid sequence to be the epitope of adiponectin

To further confirm the specific epitope recognized by KH4–8 mAb, epitope mapping was performed as described in the Methods. The epitope recognition site of the KH4–8 mAb was confirmed by peptide microarray (Fig. 4). A six–amino acid sequence (139–144), QQNHYD, from the full 244–amino acid sequence was confirmed to be the epitope of adiponectin. The amino acid sequence (91–97), PRGFPGI, was assumed to be an epitope recognized by contaminant antibody coming from the use of a recycled Protein G-Sepharose column.

### Inhibition of arthritic symptoms by monoclonal antibodies in a collagen-induced arthritis mouse model

**Fig. 5:**
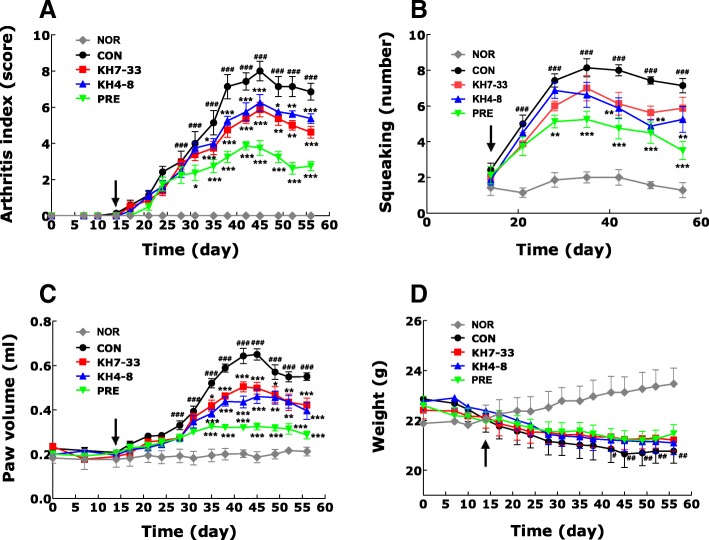
Anti-arthritic activity of monoclonal antibodies (mAbs) KH4–8 and KH7–33 in a mouse model of collagen-induced arthritis. **a** arthritis index, **b** squeaking score (a value of 0 represents no indication of pain), **c** paw volume, and (**d**) body weight, indicating the severity of arthritis in mouse limbs. The number of arthritic limbs was quantitated, and each limb was assigned a severity score of 0–4. Data indicate the number of arthritic limbs per arthritic mouse and the mean severity score of each arthritic limb. The black arrows indicate the starting day (day 15) of mAb or prednisolone administration. NOR (normal), *n* = 8; CON (negative control), KH4–8, n = 8; KH7–33, n = 8; PRE (prednisolone, positive control), *n* = 8. Values are expressed as mean ± standard error of the mean. ^###^*P* <0.001 versus the NOR group and *P <0.05, **P <0.01, ***P <0.001 versus the CON group (two-way analysis of variance followed by Bonferroni correction)

**Fig. 6:**
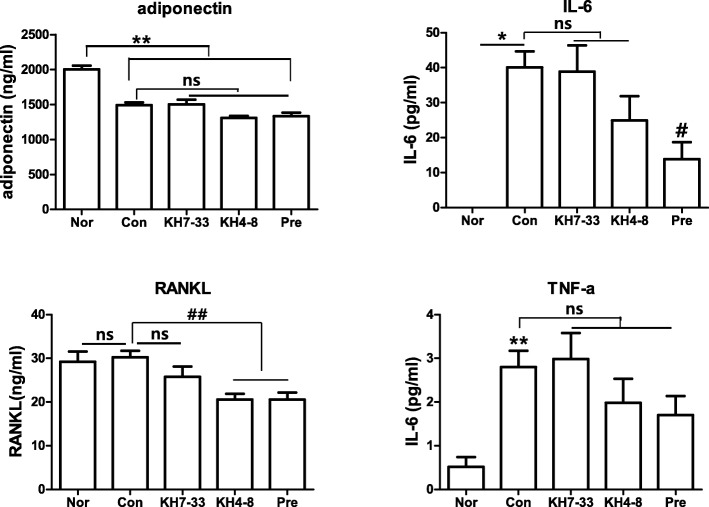
Anti-inflammatory effect of monoclonal antibodies (mAbs) KH4–8 and KH7–33 on the expression of serum pro-inflammatory cytokines of the collagen-induced arthritis mouse model. The serum levels of adiponectin, interleukin-6 (IL-6), tumor necrosis factor-alpha (TNF-α), and receptor activator of nuclear factor-kappa Β ligand (RANKL) were analyzed by using the Luminex system. Values are expressed as mean ± standard error of the mean. The expression levels of the factors were compared between groups (*n* = 8) by using the Mann–Whitney test. **P <0.01, *P <0.05 versus normal (NOR) group, and ^##^*P* <0.01, ^##^*P* <0.05 versus the control (CON) group. Abbreviations: *ns* not significant, *pre* prednisolone

**Fig. 7:**
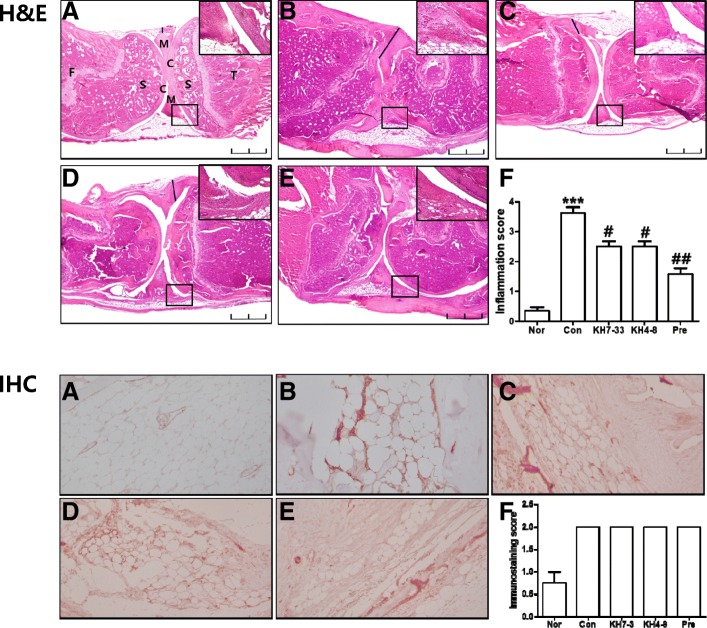
Anti-adiponectin antibodies reduce the histological signs of inflammation. The upper and lower panels present hematoxylin and eosin (H&E) staining and immunostaining against mouse adiponectin of mouse knee joints (n = 8), respectively. **a** Normal, **b** control, saline-treated arthritic, **c** KH7–33-treated arthritic, **d** KH4–8-treated arthritic, and (**e**) prednisolone-treated arthritic mice. Tissue structure was visualized by using H&E staining (original magnification, 40×). Scale bar = 2 mm. **f** Arthritic symptoms were evaluated by scoring the degree of inflammation on H&E histological sections of knee joints as described in the Methods. Small blue squares on H&E staining are magnified in the upper right corner (400×). Abbreviations: *C* cartilage, *F* femur, *M* meniscus, *S* subchondral bone, *T* tibia. In the lower panel, immunohistochemistry (IHC) reveals adiponectin expression in collagen-induced arthritis mouse joints (200×). The increased adiponectin expression level observed on IHC was not decreased by monoclonal antibody treatment. Adiponectin immunostaining score level was evaluated as described in the Methods. Results are presented as the mean of experiments (± standard error of the mean indicated by error bar) (one-way analysis of variance followed by Dunn’s multiple comparison test). ***P <0.001 versus the normal (NOR) group and ^#^*P* <0.05, ^##^*P* <0.01 versus the control (CON) group. Abbreviation: *pre* prednisolone

To test the *in vivo* anti-arthritic effect of our mAbs against adiponectin, mAbs were injected intraperitoneally into a CIA mouse model. As shown in Fig. 5, the injection of mAbs from the KH4–8 and KH7–33 clones inhibited the arthritic symptoms in CIA mice. The anti-arthritic effect of the mAbs was demonstrated by measuring the arthritis index, squeaking index, and paw volume. To further demonstrate the anti-inflammatory effect of mAbs, sera from CIA mice were analyzed for pro-inflammatory cytokines, such as adiponectin, IL-6, TNF-α, and RANKL, by using a Luminex system (Fig. 6). TNF-α and IL-6 were significantly increased in sera of CIA mice compared with that of normal mice and were slightly decreased by treatment with mAb KH4–8 or KH7–33. However, the decrease in adiponectin in CIA mice was not reversed by treatment with mAbs. In contrast, RANKL level in the sera of CIA mice did not vary compared with that of normal mice. The histology of knee joints in CIA mice further demonstrated the anti-arthritic effect of mAb KH4–8 or KH7–33. As shown in Fig. 7, joint inflammation was increased and the joint cavity was destroyed in CIA mice. Treatment with antibody KH7–33 significantly decreased both the inflammation area and degradation of the joint cavity in arthritic joints compared with those of control CIA mice. Furthermore, we investigated whether adiponectin expression was increased in the joints of CIA mice. The expression of adiponectin was increased in adipose tissue around the joints of CIA mice compared with normal mice on the basis of immunohistochemistry (Fig. 7). However, adiponectin expression increased via induction of inflammation was not decreased by treatment with the mAbs used in this experiment or anti-inflammatory agents.

## Discussion

In this study, we developed hybridomas producing mAbs against human adiponectin isoforms as potential therapeutic agents for inflammatory diseases such as RA. These mAbs were shown to recognize human adiponectin isoforms in human sera and tissues by Western blot and immunohistochemistry. Furthermore, these mAbs inhibited IL-6 and IL-8 production in HUVECs and human osteoblasts stimulated with recombinant adiponectin, suggesting that these mAbs blocked the action of adiponectin in the cells. The *in vivo* anti-arthritic effects of our mAbs were demonstrated in a CIA mouse model on the basis of the arthritis index, squeaking index, and paw volume, in agreement with the observed *in vitro* anti-inflammatory effects of the mAbs. In addition, increased levels of pro-inflammatory cytokines such as TNF-α and IL-6 in the sera of CIA mice were slightly decreased by treatment with mAbs. The anti-arthritic effects of the mAbs were also demonstrated by histology of CIA mouse knee joints. In particular, mAb KH4–8 was found to recognize a six–amino acid sequence (139–144), QQNHYD, from the full 244–amino acid sequence as the epitope of adiponectin. All of these results suggest the potential of mAbs against recombinant adiponectin as a therapeutic antibody in the treatment of RA.

For the development and production of these antibodies, hybridomas producing mAbs against recombinant human adiponectin were screened by ELISA and Western blot. IgGs from culture supernatants were purified by using a Protein G column, and the purity thereof was determined by Coomassie blue staining. The only proteins detected were the heavy and light chains of mouse IgG. The identities thereof were confirmed by using an anti-mouse IgG secondary antibody coupled to horseradish peroxidase (Additional file 1). To test cross-species reactivity, mAbs purified from three hybridoma clones—KH7–41, KH7–33, and KH4–8—were tested for their ability to detect adiponectin in rat and mouse sera. Western blots employing these mAbs showed different patterns of recognition of adiponectin in human serum, whereas the mAbs had similar recognition patterns in rat and mouse serum. KH7–41 recognized two isoforms of human adiponectin: LMW and MMW. The KH4–8 mAb recognized MMW and HMW, whereas the KH7–33 mAb recognized only one isoform of human adiponectin: MMW. These results indicate that each antibody is molecularly distinct and recognizes a different epitope of human adiponectin.

To generate adiponectin isoform-specific mAbs, adiponectin isoforms purified from serum via gel filtration may be used to immunize mice [34]. This approach can be very labor-intensive. However, this study shows that recombinant adiponectin can also be used to generate mAbs against distinct adiponectin isoforms. Based on our Western blot results, the KH4–8 mAb probably targets HMW/MMW adiponectin isoforms, which are believed to induce higher expression of inflammatory cytokines than the LMW isoform [35]. In addition, the serum levels of adiponectin isoforms change under disease conditions. For example, in anorexia nervosa, the percentage of HMW relative to total adiponectin (%HMW) is remarkably lower and the percentage of LMW relative to total adiponectin (%LMW) is significantly higher in the anorexia nervosa group compared with the control group [36]. In patients with hypertension, HMW adiponectin has been reported to be significantly lower (*P* < 0.05) and LMW adiponectin significantly higher (*P* < 0.01) than in normotensive persons [37]. The serum level of MMW adiponectin has also been shown to be decreased in endometrial cancer [38]. Adiponectin also plays a central role in obesity-related disease. HMW seems to have the predominant action in metabolic tissues. A recent report demonstrated that an increased LMW/total adiponectin ratio was associated with type 2 diabetes through a relationship to increasing insulin resistance [39]. Adiponectin can be a therapeutic target for obesity, diabetes, and endothelial dysfunction [40]. Thus, it has been suggested that targeting detrimental adiponectin isoforms while maintaining beneficial adiponectin isoforms might prevent or decrease the risk of certain diseases. In addition, mAbs against adiponectin isoforms may be used to study the distribution of adiponectin isoforms in tissues. As shown in Fig. 2, adiponectin isoforms are differentially distributed in tissues. Their differential distribution may also be involved in the pathogenesis of disease or may result from the progression of disease [41, 42]. Moreover, physical exercise induced a 21% decrease in HMW/LMW, whereas diet-induced weight loss shifted the distribution toward a higher molecular weight (42% increase in HMW/MMW) [43]. However, more accurate methods for measuring changes in adiponectin and its isoforms are needed. The mAbs used in this study have potential for measuring adiponectin variation as well as in a variety of clinical diagnostics. For example, KH4–8 mAb can be used for the diagnosis of diet-induced weight loss effects because KH4–8 mAb specifically recognizes the HMW/MMW isoform. These mAbs can also be applied for diagnosis of hypertension or inflammatory disease, which are associated with isoform distribution changes.

Adiponectin level was decreased in the serum of CIA mice, as shown in Fig. 6. However, adiponectin expression was increased in adipose tissue near arthritic joints in CIA mice. In addition, our previous studies demonstrated that adiponectin level in the joint fluid of patients with RA was greatly increased compared with that of patients with osteoarthritis [14]. All of these studies indirectly suggest that adiponectin may be more involved in joint inflammation than other tissues in the CIA mouse model. The mAbs had *in vivo* anti-arthritic effects in a CIA mouse model. In future experiments, the mAbs will be modified to a humanized antibody for human clinical trials, with the goal of using therapeutic antibodies against a specific adiponectin isoform to target specific detrimental isoforms of adiponectin in various diseases while maintaining the function of beneficial isoforms.

## Conclusions

The mAbs from hybridomas generated against a recombinant human adiponectin monomer recognized adiponectin isoforms in serum and tissue. These mAbs can potentially be developed as therapeutic or diagnostic antibodies, with the goal of targeting specific detrimental isoforms of adiponectin while maintaining the function of beneficial isoforms. Thus, mAbs against specific adiponectin isoforms could potentially be developed into therapeutic agents to treat RA or inflammatory disease.

## Additional file

Additional file 1: Screening of hybridoma clones that produced monoclonal antibodies (MAb) against a monomeric recombinant human adiponectin. (A) Supernatant (10 ml) from each clone culture was used to detect human recombinant adiponectin in each lane. Clones in lane 1, 3, and 4 were positive. (B) Positive clones were named and cultured. Culture supernatants were collected and purified by protein G-Sepharose column chromatography as described in Materials and Methods. The purity was determined by Coomassie blue staining after PAGE. (C) The purified protein bands were confirmed as IgGs by Western blot using anti-mouse IgG-HRP.(JPG 34 kb)

## Abbreviations

ANOVA: Analysis of variance
CFA: Complete Freund’s adjuvant
CIA: Collagen-induced arthritis
CII: Collagen type II
ELISA: Enzyme-linked immunosorbent assay
HMW: High molecular weight
HUVEC: Human umbilical vein endothelial cell
IL: Interleukin
LMW: Low molecular weight
mAb: Monoclonal antibody
MMW: Middle molecular weight
OD: Optical density
RA: Rheumatoid arthritis
RANKL: Receptor activator of nuclear factor-kappa Β ligand
SDS-PAGE: Sodium dodecyl sulfate-polyacrylamide gel electrophoresis
TNF: Tumor necrosis factor
