# Supplementary figures and images for: Potential therapeutic antibodies targeting specific adiponectin isoforms in rheumatoid arthritis
Source: Arthritis Res Ther. 2018 Oct 30;20:245. doi: 10.1186/s13075-018-1736-3 (PMC6235220; doi:10.1186/s13075-018-1736-3)

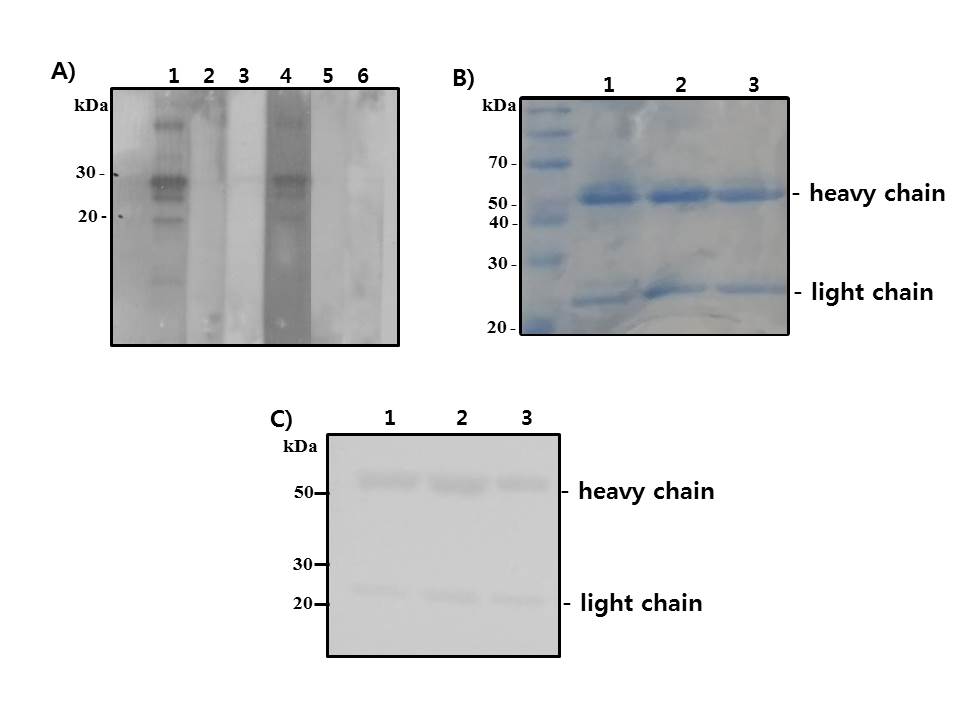

Supplement: Supplementary file 1 — Screening of hybridoma clones that produced monoclonal antibodies (MAb) against a monomeric recombinant human adiponectin. (A) Supernatant (10 ml) from each clone culture was used to detect human recombinant adiponectin in each lane. Clones in lane 1, 3, and 4 were positive. (B) Positive clones were named and cultured. Culture supernatants were collected and purified by protein G-Sepharose column chromatography as described in Materials and Methods. The purity was determined by Coomassie blue staining after PAGE. (C) The purified protein bands were confirmed as IgGs by Western blot using anti-mouse IgG-HRP.(JPG 34 kb) [file 13075_2018_1736_MOESM1_ESM.jpg]
